# A modeling-based framework to evaluate forgiveness of tuberculosis treatment in a BALB/c relapsing mouse model

**DOI:** 10.1128/aac.01109-25

**Published:** 2026-01-15

**Authors:** Sylvie Sordello, Laure Brock, Alessia Tagliavini, Denise Federico, Xavier Boulenc, Marco Pergher, Emilie Huc Claustre, Darren Metcalf, Nicholas D. Walter, Gregory T. Robertson, James Clary, Alexander Berg, Khisi Mdluli, David Hermann, Debra Flood, Anna M. Upton

**Affiliations:** 1 Translational Biology, Infection Diseases, Evotec France, Toulouse, France; 2 Pharmacometrics, Aptuit (an Evotec Company), Verona, Italy; 3 DMPK, Evotec Infectious Diseases Lyon, Lyon, France; 4 DMPK, Evotec France, Toulouse, France; 5 Rocky Mountain Regional VA Medical Center 19982 , Aurora, Colorado, USA; 6 Division of Pulmonary Sciences and Critical Care Medicine, University of Colorado Anschutz Medical Campus, Aurora, Colorado, USA; 7 Consortium for Applied Microbial Metrics, Aurora, Colorado, USA; 8 Mycobacteria Research Laboratories, Department of Microbiology, Immunology and Pathology, Colorado State University 3447, Fort Collins, Colorado, USA; 9 Allucent (US) LLC, Cary, North Carolina, USA; 10 IceBerg Consulting, West Fargo, North Dakota, USA; 11 Gates Medical Research Institute 533273 , Cambridge, Massachusetts, USA; 12 Gates Foundation 11037, Seattle, Washington, USA; 13 Evotec USA inc., New Jersey, USA; City St George's, University of London, London, United Kingdom

**Keywords:** anti-tubercular, RS ratio, forgiveness, TB relapse mouse model, tuberculosis

## Abstract

Tuberculosis (TB) remains a leading cause of death due to an infectious agent. Adherence to long and complex TB treatments is supported by methods including directly observed therapy. The negative impact of missed drug doses on clinical outcomes is well established, highlighting both the importance of adherence support and methods to quantify the ability of a regimen to continue exerting a biologic effect during gaps in dosing known as treatment “forgiveness.” To explore the value of the BALB/c relapsing mouse model of TB in evaluating treatment forgiveness, we assessed the impact of weekend dose holidays on the bactericidal efficacy, including CFU and RS ratio reduction and sterilizing efficacy, of RHZE/RH and BPaMZ. The cure/relapse data from this study, plus multiple historical studies, were used to identify a nonlinear mixed-effects Emax model that was then used to estimate time to cure 50% and derive time to cure 90% of mice (T90). The expected time-dependent bactericidal activity and reductions in RS ratio were observed for both treatments, with more rapid decreases for the BPaMZ groups. The weekend dosing holiday significantly decreased reductions in lung CFU and RS ratio earlier in RHZE/RH treatment, but no such effect was observed for BPaMZ. Similarly, the predicted T90 was significantly greater for RHZE/RH (but not BPaMZ), with weekend doses omitted. No major drug exposure difference was observed between the two dosing schedules. Our results suggest that BPaMZ is more forgiving of missed doses than RHZE/RH and demonstrate the utility of this methodology to support the evaluation of TB treatment forgiveness.

## INTRODUCTION

Tuberculosis (TB) is the leading cause of death from an infectious agent (1). A major obstacle to TB control is poor adherence to current treatments, which are lengthy, complicated, and often poorly tolerated. Adherence support is also suboptimal: directly observed therapy (DOT) is resource-intensive and typically not performed during weekends. While missed weekend doses present one potential source of nonadherence, the importance of daily dosing has been demonstrated in several analyses (2–6) .

New treatment strategies aim to be shorter, simpler, and better tolerated, but are likely to remain lengthy (2, 7, 8). For this reason, a 2023 update to the World Health Organization (WHO) target regimen profiles (TRPs) for TB treatments introduced a new criterion, namely, forgiveness. Forgiveness is defined operationally as “the degree to which regimen efficacy is unaffected by suboptimal adherence” and is thought to be influenced by the pharmacokinetic/pharmacodynamic (PK/PD) profile of drugs in the regimen, as well as the post-antibiotic effect (PAE), which is the lag after drug exposure before bacteria recover and resume growth (9–11). Forgiveness of TB drug regimens is difficult to explore in humans for ethical, technical, and clinical reasons (9). Therefore, the WHO encouraged assessment in preclinical models.

Here, we aimed to assess the utility of the well-established BALB/c relapsing mouse model of TB (RMM) (12–19) in the evaluation of forgiveness by comparing the efficacy of two clinically relevant TB drug regimens when given daily or with a weekend dosing holiday as a proxy for missed doses. As test regimens, we selected the standard drug-susceptible TB regimen RHZE/RH and the shorter experimental SimpliciTB regimen BPaMZ. We adopted recently reported approaches to experimental design and mathematical modeling of data (19–21) to enable derivation of time to cure 90% of mice (T90) based on microbiological cure/relapse data. We included assessment of the novel PD marker RS ratio (22–24), which quantifies *M.tb* rRNA synthesis, to determine whether this marker, which provides a measure of bacterial activity, would enhance the usefulness of this animal model in studies of TB drug regimen forgiveness.

## RESULTS

### Impact of 5/7 versus 7/7 dosing on bactericidal and RS ratio activity

**Fig 1:**
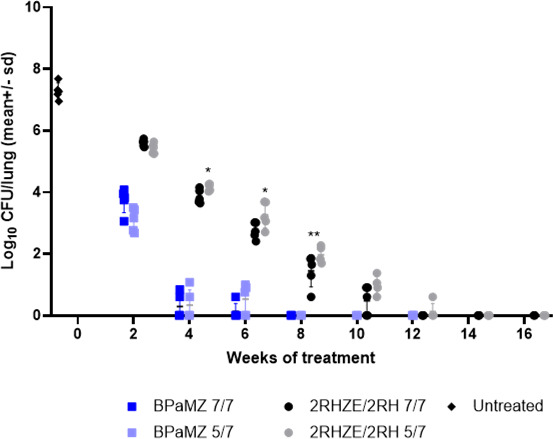
Mouse lung CFU following treatment with 2RHZE/2RH and BPaMZ dosed 5/7 and 7/7 days per week. Whole-lung CFU of BALB/c mice, intranasally infected with *M.tb* H37Rv, after different durations of oral treatment with 2RHZE/2RH or BPaMZ, dosed 5/7 or 7/7 days per week. Treatments were initiated 2 weeks post-infection. Statistical test results for dosing-days comparison on Log_10_ CFU/lung are indicated as follows: *: *P* < 0.05; **: *P* < 0.01.

**Fig 2:**
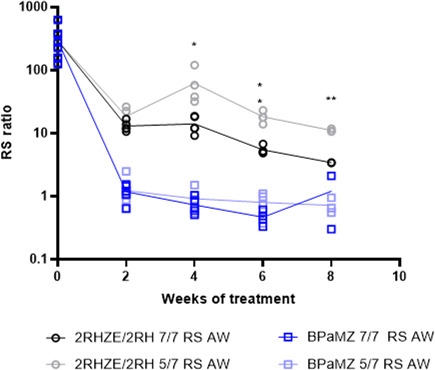
RS ratio in mouse lungs following treatment with 2RHZE/2RH and BPaMZ dosed 5/7 and 7/7 days per week. RS ratio in the lungs of BALB/c mice, intranasally infected with *M.tb* H37Rv, after different durations of oral treatment with 2RHZE/2RH or BPaMZ, dosed 5/7 or 7/7 days per week. Treatments were initiated 2 weeks post-infection. At different time points, lungs were collected for RS ratio quantification. Analysis was performed on one-third of the lung. Statistical test results for dosing-days comparison on RS ratio are indicated as follows: *: *P* < 0.05; **: *P* < 0.01.

One day (Day 13) and 14 days (Day 0) post-infection, mouse lung bacterial burdens were 4.5 ± 0.09 and 7.28 ± 0.26 Log_10_ CFU/lung, respectively (mean ± SD). The drug combinations, BPaMZ and 2RHZE/2RH, were administered either 5/7 or 7/7 days per week according to the study design illustrated in Table S1 and Fig. S1. Lung bacterial burdens declined significantly in a time-dependent manner following oral treatment with BPaMZ or RHZE/RH given 5/7 or 7/7 days per week. BPaMZ had greater bactericidal activity than 2RHZE/2RH, achieving culture negativity in all mice after 8 weeks of treatment (Fig. 1). The RS ratio also declined significantly faster for BPaMZ than for 2RHZE/2RH, and a more profound reduction was seen with the former combination (Fig. 2). No significant differences were observed in the overall time to culture negativity in mice given BPaMZ 5/7 or 7/7 days per week. In contrast, 12 or 14 weeks of 2RHZE/2RH were required to achieve negative lung cultures following 7/7 or 5/7 dosing, respectively. For 2RHZE/2RH dosed 7/7, mice had a significantly lower lung CFU burden at 4, 6, and 8 weeks of treatment than mice allowed weekend drug holidays (5/7) (Fig. 1). There was no significant CFU difference between 7/7 and 5/7 dosing at weeks 2 and 10. Similarly, mice dosed with 2RHZE/2RH daily had significantly greater suppression of the RS ratio at weeks 4, 6, and 8 relative to mice allowed weekend holidays (Fig. 2), while no such difference was observed in the BPaMZ-treated mice for either CFU (Fig. 1) or RS ratio (Fig. 2).

### Change in CFU and RS ratio during a short treatment interruption

**Fig 3:**
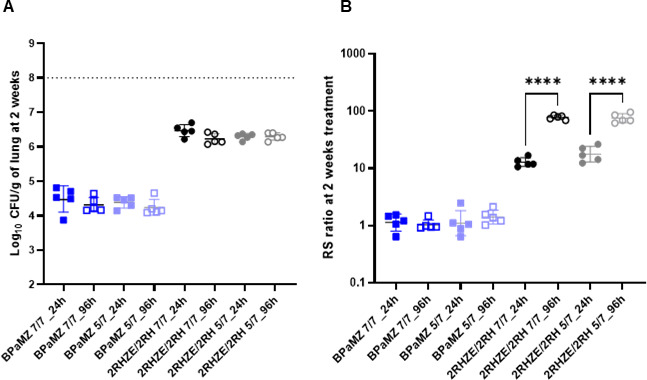
Mouse lung CFU (**A**) and RS ratio (**B**) measured 24 h or 96 h post-last dosing at the end of 2 weeks of treatment. Lungs of BALB/c mice, intranasally infected with *M.tb* H37Rv, after 2 weeks of oral treatment with 2RHZE/2RH or BPaMZ, dosed 5/7 or 7/7 days per week, initiated 2 weeks post-infection, were split in two-thirds for CFU analysis and one-third for RS ratio analysis. Lungs were collected 24 h or 96 h post-last dosing. Statistical test results for dosing-days comparisons on Log_10_ CFU/lung are indicated as follows: ****: *P* < 0.0001.

To explore the impact of a short interruption of treatment on PD parameters, we compared CFU and RS ratio in lungs collected 24 h or 96 h post-last dosing following 2 weeks of treatment. There was no significant difference in CFU between a 24-hour (1-day) or 96-hour (4-day) recovery period after stopping treatment with BPaMZ or RHZE (Fig. 3A). For RHZE, the RS ratio increased significantly during this short period following end of treatment. Between post-treatment days 1 and 4, the median RS ratio rose from 17 to 75 with 5/7 dosing and from 12 to 80 with 7/7 dosing (Fig. 3B). By contrast, there was no significant change in the RS ratio between post-treatment days 1 and 4 for BPaMZ.

### Significant increase in time to 90% cure (T90), derived using an Emax model, for 2RHZE/2RH, but not BPaMZ, with a weekend dosing holiday

**Fig 4:**
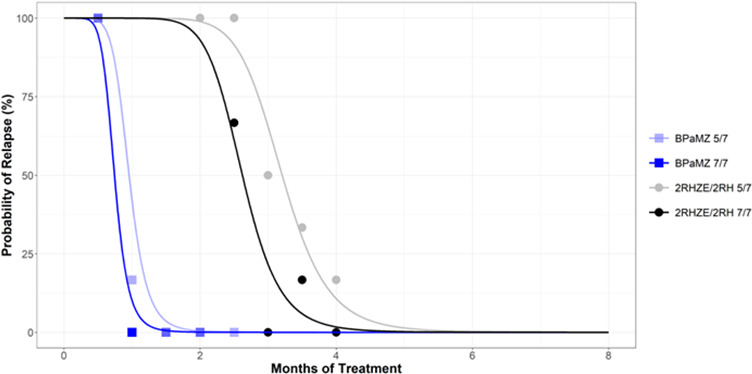
Probability of relapse with treatment duration for BALB/c mice treated with 2RHZE/2RH and BPaMZ. Sterilization curves indicating the probability of relapse over treatment time, constructed by fitting observed relapse data (Table 2) to an Emax model developed using a large historical RMM data set. Observed relapse data are indicated for each test regimen using open symbols and crosses. The time to 50% cure/relapse is estimated from these curves, and the time to 90% cure (i.e., 10% relapse) is derived utilizing time to 50% cure estimates together with steepness of the curve (gamma), as explained in Methods.

To establish the relationship between treatment time and durable cure for the tested regimens, we quantified the proportion of mice that were culture-positive (i.e., relapse) 12 weeks after the end of treatment, then performed model-based analysis to estimate the time to 50% cure and derive the time to 90% cure (T90). For BPaMZ-treated groups, very few mice relapsed following 4 weeks of treatment, whereas 100% relapsed following 2 weeks of treatment. No relapse events were recorded for any BPaMZ-treated mice after at least 6 weeks of treatment (Table 1). In contrast, at least 16 weeks of 2RHZE/2RH was required to achieve low or no relapse (Table 1). Based on a logistic Emax model developed from observed cure/relapse percentages (Fig. 4), derived T90s of 1.29 and 4.03 months were obtained for BPaMZ and 2RHZE/2RH dosed 5/7, respectively. For 7/7 dosing regimens, the derived T90s were 1.00 months for BPaMZ and 3.30 months for 2RHZE/2RH (Table 2). While a significant difference of 0.7 months was observed between the T90s for RHZE/RH, with 5/7 versus 7/7 dosing (*P* = 0.025), there was no significant difference between the T90s for the 5/7 or 7/7 dosed BPaMZ regimens (0.3 months, *P* = 0.110) (Table 2).

**TABLE 1:** Percentage of mice with evidence of relapse for each combination (positive culture mice*/total number of mice)

Drug regimen	Weeks of treatment prior to the 12-week post-treatment relapse phase							
	2W	4W	6W	8W	10W	12W	14W	16W
BPaMZ 7/7	100% (6/6)	0% (0/6)	0% (0/6)	0% (0/5)	NA	NA	NA	NA
BPaMZ 5/7	100% (6/6)	16.6% (1/6)	0% (0/6)	0% (0/6)	0% (0/6)	NA	NA	NA
2RHZE/2RH 7/7	NA^*a*^	NA	NA	100% (6/6)	66.6% (4/6)	0% (0/6)	16.6% (1/6)	0% (0/6)
2RHZE/2RH 5/7	NA	NA	NA	100% (6/6)	100% (6/6)	50% (3/6)	33.3% (2/6)	16.6% (1/6)

^*a*^ NA, not applicable.

**TABLE 2:** Estimated time to 50% cure (T50) and derived time to 90% cure (T90) values for test combinations

	T50 estimated (months)	T90 derived (months)	Delta T90 (5/7 vs 7/7) (months)	Delta T90 *P*-value
BPaMZ 7/7	0.74	1.00	0.29	0.110
BPaMZ 5/7	0.96	1.29		
2RHZE/2RH 7/7	2.62	3.30	0.74	0.025
2RHZE/2RH 5/7	3.21	4.03		

### A limited impact of dosing 5/7 versus 7/7 days per week on drug exposures is only evident for Pa and M

**Fig 5:**
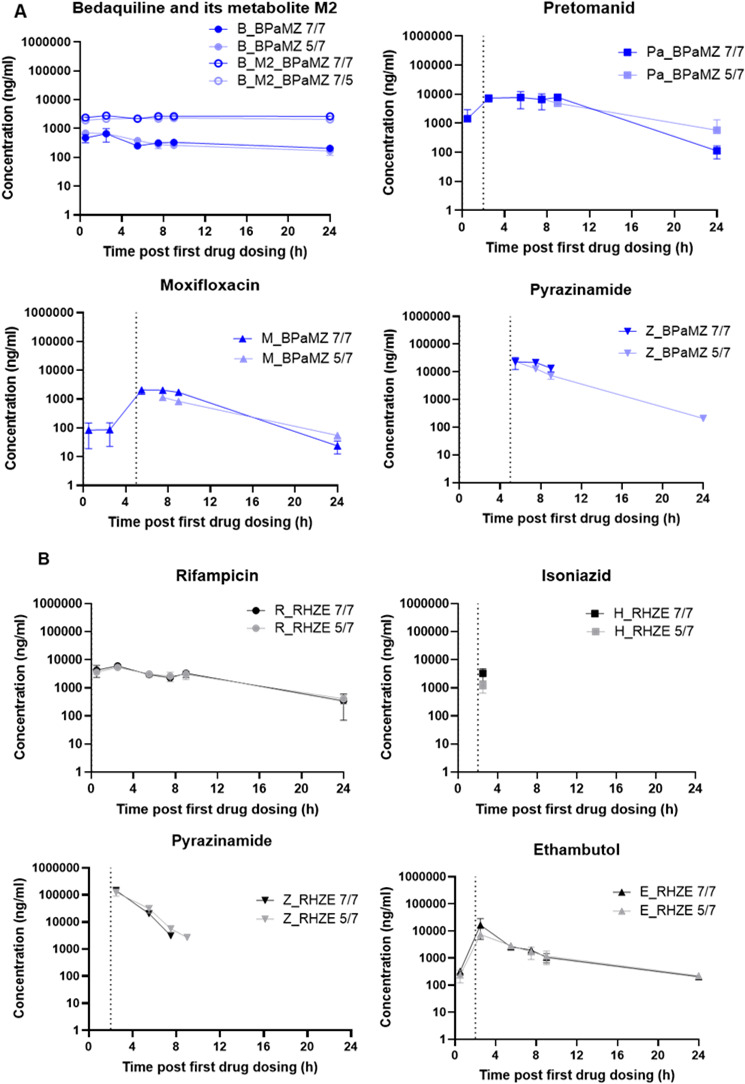
Individual blood PK after 8 weeks of treatment. Blood samples were collected on the 5th day of the last week of treatment. For each combination (six mice per group), samples were taken at 0.5, 2.5, 5.5, 7.5, 9, and 24 h post-first compound dosing. Individual drug exposure was determined in BPaMZ (**A**) and RHZE (**B**) after 5/7 or 7/7 dosing. Note: The dotted line in the graph corresponds to the time of dosing start of the corresponding drug.

Following 8 weeks of treatment, drug exposures were assessed for all treatment groups. The observed exposures were similar to each other for all individual drugs, when given 5/7 or 7/7 days per week, with the exception of Pa and M dosed within BPaMZ (Fig. 5A and B). Whereas Pa and M, following the 7/7 administration schedule, were still detectable 24 h after the previous dosing, both drugs were below the limit of quantification (LOQ) when given 5/7 (Fig. 5A). Despite these differing C_trough_ concentrations, the post-administration concentrations in blood for Pa and M were similar between the two dosing schedules (Fig. 5A). In RHZE, no significant differences in exposures were observed between the two dosing schedules. However, it should be noted that H was detected at only one time point (Fig. 5B).

## DISCUSSION

This optimized study design supported testing the impact of missed TB drug doses of two benchmark drug combinations in a well-established mouse model of TB, where missed doses were simulated using a weekend dosing holiday. The absolute and relative time-dependent bactericidal activity (measured by CFU) and impact on RS ratio of the 2RHZE/2RH and BPaMZ regimens given 5/7 days per week were consistent with previous published data (14–18, 22, 23). Although derived via differing methodology, the derived T90s of 4.03 and 1.29 months the 2RHZE/2RH and BPaMZ given 5/7 days per week, respectively, are also similar to published values (19, 20).

The statistically significant impact of the weekend dosing holiday on the BALB/c mouse T90 for the 2RHZE/2RH treatment is consistent with analyses of clinical data indicating that the current rifampin-based regimen used worldwide has low forgiveness for non-adherence or missed doses (2, 4). The low forgiveness of this regimen has important implications for TB care as well as clinical trial design. While adherence support innovations are advancing, such tools can be limited by issues of scale-up, generalizability, and cost. A more durable and patient-centered solution is the targeted development of regimens composed of drugs that demonstrate high forgiveness. These would accommodate less than perfect adherence patterns in the field without meaningful penalty to efficacy.

The increase in T90 observed for 2RHZE/2RH was correlated with differences in both the kinetics of bactericidal responses (lung CFU reduction) and the reduction in RS ratio during the first 8 weeks. Our evaluation of a brief treatment interruption after 2 weeks of treatment confirmed previous observations that the RS ratio “rebounds” when RHZE is stopped, suggesting the onset of *Mycobacterium tuberculosis* “physiologic recovery” very soon after treatment cessation, which is not observable via assessment of CFU (25). No differences were observed for any of the tested PD parameters for BPaMZ between the two dosing schedules, nor was any RS ratio “rebound” seen, which is consistent with the apparent forgiveness of BPaMZ and might be attributable to either the PK profile of B (26–28) or to the distinct pattern of bacterial physiological injury and adaptation caused by B-based regimens (i.e., PAE) (29), the two factors thought to influence treatment forgiveness.

The PK profiles of drugs in these regimens were analyzed at steady state, and drug levels found in blood were generally consistent with literature values (26–28, 30–32). The observed differences in drug exposures between 2RHZE/2RH given 5/7 versus 7/7 days per week do not readily explain the impact of the weekend holiday on treatment efficacy outcomes. The exposures of all drugs in this combination were similar at the time points evaluated (after the 5^th^ dose given in the 8^th^ week of dosing), which is assumed to represent steady state. However, we cannot rule out the differences in exposure dynamics that may have occurred during weekend breaks or earlier in the course of treatment. On the other hand, modest differences were observed for exposures of Pa and M between 5/7 and 7/7 dosing schedules, but this did not have a significant impact on the bactericidal activity, RS ratio reduction over time, or T90 for BPaMZ. As for 2RHZE/2RH, exposure dynamics over treatment time were not evaluated, nor was the observed drug clearance over the weekend dosing holiday. In contrast, B and its active metabolite M2 demonstrated a long half-life (Fig. 5) and a slow release from tissues and lesions after treatment cessation (28) (supplementary data Fig. S2), which may be sufficient to reduce any effects of exposure differences for Pa and M between the 7/7 and 5/7 dosing schedules.

It is interesting to note that, based on all pharmacological parameters measured, the two-day dosing holiday (i.e., 5/7 dosing) appears to significantly impact only the less effective 2RHZE/2RH drug combination, even though steady-state drug exposures at the end of 8 weeks of treatment did not appear to be changed by a weekend dosing holiday. On the other hand, the RS ratio increase observed within a short period following the cessation of RHZE treatment may indicate rapid physiological recovery from drug injury of bacteria experiencing drug holidays with 2RHZE/2RH treatment only. It is possible that more effective drug regimens, such as BPaMZ, which rapidly and profoundly reduce lung CFU and RS ratio, are more forgiving due to the injury they cause to pathogens (i.e., PAE), either in addition to or rather than their overall PK characteristics. Further studies to evaluate this proposal are warranted.

The data from the present study support the importance of daily dosing of 2RHZE/2RH to optimize treatment efficacy and suggest potential utility of the BALB/c mouse model of TB for evaluating forgiveness of novel TB drug combinations. The forgiveness of BPaMZ with respect to the treatment of TB patients is not yet known.

Like all models, the BALB/c RMM has limitations, including pathology that does not include all lesion types seen in TB patients, such as those exhibiting caseous necrosis and cavitation. For this reason, the extent to which findings from the BALB/c RMM translate to the clinic is hard to predict, and it is likely that evaluation of forgiveness will require the development of additional tools or models. However, it should be noted that more favorable outcomes were also observed in human TB patients in the clinic when rifamycin-based regimens were administered 7 out of 7 versus 6 out of 7 days per week (4). The PK analyses conducted in this study were limited and preclude a detailed understanding of PK dynamics throughout dosing and treatment intervals and thus should be considered with some degree of caution in the context of their implications for the relative PK of these TB drug regimens with respect to forgiveness. Generation of a richer data set together with population pharmacokinetic (POPPK) modeling would provide more detailed insights. Similarly, further studies to assess forgiveness of other TB drug regimens for which PAE has been characterized would improve the understanding of the role of PAE, as well as of the utility of the BALB/c RMM to predict clinical forgiveness.

Notwithstanding these limitations, the more limited impact of weekend dosing holidays on the derived T90 of BPaMZ versus RHZE/RH, as observed using this mouse TB model, suggests that forgiveness or impact of missed doses may be less relevant to clinical outcomes for drug combinations that include drugs with characteristics similar to BPaMZ rather than RHZE/RH. These characteristics may include specific PK characteristics in addition to the ability to promote profound pathogen injury (i.e., long PAE) and could be used to develop new TB regimens that address the WHO TRP for treatment forgiveness.

## MATERIALS AND METHODS

### Animals

Six-week-old female BALB/cJRj from Janvier Laboratories were used in these studies. Mice were group housed in bioconfined cages (Isocage, Tecniplast) under a 12 h light: 12 h dark with free access to filtered water and a standard rodent diet (AO4C, Safe, France). An ambient temperature of 22 ± 2°C, a relative humidity of 55 ± 10%, and a negative pressure of −20 Pa were maintained throughout the study. All mice were allowed to acclimatize to their new environment for at least 5 days.

### Drug formulations and dosing strategies

Drugs were acquired and formulations prepared for dosing as follows: Bedaquiline (B, LTK Laboratories) was formulated in 20% 2-hydroxypropy-β-cyclodextrin for dosing at 25 mg/kg; Pretomanid (Pa, Chemshuttle) was formulated in 10% hydroxy-propyl-beta-cyclodextrin and 2% soybean lecithin for dosing at 100 mg/kg; Moxifloxacin (M, LTK Laboratories) and pyrazinamide (Z, Sigma) were co-formulated in water for dosing at 100 mg/kg and 150 mg/kg, respectively. Rifampicin (R, Sigma) was prepared in water for dosing at 10 mg/kg; Isoniazid (H, Sigma), pyrazinamide (Z, Sigma), and ethambutol (E, Sigma) were co-formulated in water for dosing at 10, 150, and 100 mg/kg, respectively. For the BPaMZ groups, B and Pa were administered individually, then co-formulated MZ was dosed. For 2RHZE/2RH groups, for the first 2 months, R was administered individually, then co-formulated HZE was dosed. For the following 2 months, R and H were dosed individually. Mice were rested 2 h between doses.

### Relapsing mouse model study design

Following the principles described in Mourik et al. (19) and utilizing the curated data set and model established in Berg et al. (21), a simulation-estimation study was performed as reported in Clary et al. (33), whereby a series of “virtual” RMM studies with alternative designs (e.g., different numbers of mice, mice per time point, and numbers of time points) were simulated to generate relapse outcomes from relapse vs treatment duration curves for control and hypothetical regimens. The resulting relapse events were analyzed by fitting the simulated data using a logistic modeling approach analogous to that described herein (33). The estimated model parameters were compared with the known input values to assess bias and precision of competing study designs in the estimation of relapse curves. These outputs were used to compare the performance of the virtual study designs and ultimately informed the selection of the study design described in Fig. 1 and in Fig. S1 and Table S1 .

In addition, to enable assessment of bactericidal activity over time for each combination and treatment regimen, mice were added to allow either lung bacterial load enumeration or measurement of changes in RS ratio biomarker immediately after completion of each treatment duration. PK analysis was also performed after 8 weeks of treatment to assess related drug exposures at steady state.

Mice were infected with *Mycobacterium tuberculosis* (*M.tb*) H37Rv stock solution prepared at exponential growth phase in 7H9 medium/10% OADC (oleic acid-albumin dextrose-catalase)/15% glycerol. At day −14, mice were anesthetized with 2.5% isoflurane in 97.5% oxygen and intranasally infected with 50 µL of *M.tb* H37Rv at an inoculum level of 4.5 Log_10_ CFU/mouse. Treatment started at day 0, 14 days post-infection. The drug combinations, BPaMZ and 2RHZE/2RH, were dosed daily per oral gavage either 5/7 or 7/7 days per week. Following the designated treatment period, mice were sacrificed 24 h post-last dosing. For relapse assessment, treatment was terminated at the end of each dosing period (i.e., 2, 4, 6, 8, 10, 12, 14, or 16 weeks of treatment, respectively), and lungs were collected 12 weeks after the end of treatment (end of the relapse/cure phase).

Lungs were collected, and procedures were conducted for evaluation of endpoints as follows. For assessment of lung CFU and RS ratio, lung collection was performed as follows: the right upper lung lobes (one-third of lung) were dissected and snap-frozen and kept at −80°C. The remaining left lung, lower right, and accessory lobes (two-thirds of lung) from this mouse were weighed and processed for bacterial enumeration. For assessment of relapse, whole lungs were collected from mice after 12 weeks off treatment. They were harvested, weighed, and processed for bacterial enumeration. In all cases, for bacterial enumeration, lung samples were homogenized and plated undiluted or serially diluted on 7H11-OADC + 0.4% activated charcoal plates and incubated at 37°C for CFU quantification.

### PK analysis

After 8 weeks of treatment (after the 5^th^ day of dosing of the 8^th^ week), for each combination (6 mice per group), blood was collected from the tail vein at 0.5, 2.5, 5.5, 7.5, 9, and 24 h post-first compound in the regime (B or R) (see dosing strategy). At the terminal time point, blood was collected by cardiac puncture for plasma preparation, and lungs were collected at 2.5 h (three mice) and 24 h (three mice) post-first compound dosing. Plasma was prepared and stored at −80°C until analysis. Lungs were collected, weighed, and stored at −80°C until processing for lung concentration analysis. After blood, plasma, and lung processing, each compound, including the M2 metabolite of B (N-mono-desmethyl metabolite, -M2), was quantified using liquid chromatography-tandem mass spectrometry (LC-MS/MS) methods.

Briefly, blood, plasma, or lung homogenate extracts were diluted with extracted control matrix as appropriate for the calibration range used. The supernatant (75 µL) was diluted 1:1 with 75 µL of water (acetonitrile for pyrazinamide, isoniazid, ethambutol, and rifampicin) prior to analysis. Bespoke chromatographic conditions were set up for: (i) B, B-M2, Pa, and M, using phase A: water with 0.1% formic acid and phase B: acetonitrile with 0.1% formic acid; and (ii) Z, H, R, and E, using phase A: water with 0.5% formic acid and phase B: acetonitrile with 0.5% formic acid. Liquid chromatographic gradients (phase A versus phase B) were applied for these two groups of compounds. The following mass spectrometry conditions were used for analysis of the compounds:

- Ionization mode : ESI positive mode
- Capillary voltage : 3 kV
- Desolvation temperature : 600°C

The LC-MS parameters (transition) are reported in the Table S2. The LOQ of each compound is given in Table S3.

### RS ratio measurement

Lung samples that were collected and snap-frozen in Precellys tubes underwent two-step homogenization and bead-beating, quantitative RNA extraction, fluorometric quantification, quality assessment via electrophoresis, reverse transcription, qPCR, and digital PCR according to the standardized workflow. This workflow is accompanied by analysis of multiple process and procedural control specimens described by Walter et al. (22). The rRNA synthesis ratio (RS ratio) was calculated as follows: unstable rRNA/stable rRNA = EST1/16S rRNA × 10,000 (22).

### Logistic Emax model

A Logistic Emax model, based on a nonlinear mixed-effects approach, was applied to relapse data obtained from the current study plus historical ones, pooling together data of the same treatment collected from different studies (i.e., within the historical and current data sets). The majority of the historical data consisted of the data set used to develop the logistic regression model reported by Berg et al. (21). In addition, historical data generated at Evotec were included. This consisted of relapse data generated for the reference TB drug combinations BPaMZ, RHZ/RH, BPaL, and PaMZ (where L stands for Linezolid) with historical design (18).

The model used is based on that described in Clary et al. (33), with the exception of the random effect on γ (i.e., η2), which is not considered in this work since its addition to the model did not significantly improve model performance. For each regimen, γ and T50 parameters were estimated and then used to calculate the related time to 90% cure (T90), according to the following formula:


(Population) $$T_{90_{i}}=10^{log10\left({T_{50}}_{i}\right)+(\frac{1}{{\gamma `}_{i}}log10\left(\frac{90}{100-90}\right))}$$

(Individual) $$T_{90_{ik}}=10^{log10\left({T_{50}}_{ik}\right)+(\frac{1}{{\gamma `}_{i}}log10\left(\frac{90}{100-90}\right))}$$

Regimens (in the historical data set) that share the same drugs but were administered at different dose levels or with differing dose schedules were parametrized with different T50 parameters, while assuming the same γ value (i.e., steepness of the relapse-time curve). This approach limits the number of parameters to be estimated, thus overcoming identifiability issues. The model was developed using NONMEM 7.5.1, and data handling was performed through SAS 9.4.

### Statistical analysis

CFU/lung was Log_10_-transformed before analysis. Zero CFU counts were replaced with 1 prior to transformation to allow calculation. The effect of dosing schedule 5/7 and 7/7 on number of CFU in lung and on RS ratio results was evaluated for BPaMZ and 2RHZE/2RH considering the period of treatment using a two-way ANOVA followed by LSD Fisher test. The effect of the dosing schedule on derived T90 was compared using a Z-test. *P*-values <0.05 were considered to be statistically significant.

## SUPPLEMENTAL MATERIAL

Supplemental material aac.01109-25-s0001.docx Tables S1, S2 and S3; Fig. S1 and S2.

ASM does not own the copyrights to Supplemental Material that may be linked to, or accessed through, an article. The authors have granted ASM a non-exclusive, world-wide license to publish the Supplemental Material files. Please contact the corresponding author directly for reuse.

## References

[1] World Health Organization. 2024 Global tuberculosis report 2024 WHO, Global tuberculosis report 2024

[2] Alipanah N, Jarlsberg L, Miller C, Linh NN, Falzon D, Jaramillo E, Nahid P. 2018. Adherence interventions and outcomes of tuberculosis treatment: a systematic review and meta-analysis of trials and observational studies. PLoS Med 15:e1002595. doi:10.1371/journal.pmed.1002595 29969463 PMC6029765

[3] Cadosch D, Abel Zur Wiesch P, Kouyos R, Bonhoeffer S. 2016. The role of adherence and retreatment in de novo emergence of MDR-TB. PLoS Comput Biol 12:e1004749. doi:10.1371/journal.pcbi.1004749 26967493 PMC4788301

[4] Imperial MZ, Nahid P, Phillips PPJ, Davies GR, Fielding K, Hanna D, Hermann D, Wallis RS, Johnson JL, Lienhardt C, Savic RM. 2018. A patient-level pooled analysis of treatment-shortening regimens for drug-susceptible pulmonary tuberculosis. Nat Med 24:1708–1715. doi:10.1038/s41591-018-0224-2 30397355 PMC6685538

[5] Chang KC, Leung CC, Yew WW, Chan SL, Tam CM. 2006. Dosing schedules of 6-month regimens and relapse for pulmonary tuberculosis. Am J Respir Crit Care Med 174:1153–1158. doi:10.1164/rccm.200605-637OC 16908866

[6] Vashishtha R, Mohan K, Singh B, Devarapu SK, Sreenivas V, Ranjan S, Gupta D, Sinha S, Sharma SK. 2013. Efficacy and safety of thrice weekly DOTS in tuberculosis patients with and without HIV co-infection: an observational study. BMC Infect Dis 13:468. doi:10.1186/1471-2334-13-468 24099345 PMC3852441

[7] Stagg HR, Thompson JA, Lipman MCI, Sloan DJ, Flook M, Fielding KL. 2023. Forgiveness is the attribute of the strong: nonadherence and regimen shortening in drug-sensitive tuberculosis. Am J Respir Crit Care Med 207:193–205. doi:10.1164/rccm.202201-0144OC 35952354 PMC9893326

[8] Fox WS, Strydom N, Imperial MZ, Jarlsberg L, Savic RM. 2023. Examining nonadherence in the treatment of tuberculosis: the patterns that lead to failure. Br J Clin Pharmacol 89:1965–1977. doi:10.1111/bcp.15515 36036095

[9] World Health Organization. 2023. Target regimen profiles for tuberculosis treatment. Available from: https://www.who.int/publications/i/item/9789240081512 10.2471/BLT.24.291881 PMC11276158 39070602

[10] MacKenzie FM, Gould IM. 1993. The post-antibiotic effect. J Antimicrob Chemother 32:519–537. doi:10.1093/jac/32.4.519 8288494

[11] Pai MP, Cottrell ML, Kashuba ADM, Bertino JS Jr. 2015. Pharmacokinetics and Pharmacodynamics of Anti-infective Agents, p 252–262. In Mandell, Douglas, and Bennett’s Principles and Practice of Infectious Diseases

[12] Cevik M, Thompson LC, Upton C, Rolla VC, Malahleha M, Mmbaga B, Ngubane N, Abu Bakar Z, Rassool M, Variava E, et al.. 2024. Bedaquiline-pretomanid-moxifloxacin-pyrazinamide for drug-sensitive and drug-resistant pulmonary tuberculosis treatment: a phase 2c, open-label, multicentre, partially randomised controlled trial. Lancet Infect Dis 24:1003–1014. doi:10.1016/S1473-3099(24)00223-8 38768617

[13] Xu J, Li SY, Almeida DV, Tasneen R, Barnes-Boyle K, Converse PJ, Upton AM, Mdluli K, Fotouhi N, Nuermberger EL. 2019. Contribution of pretomanid to novel regimens containing bedaquiline with either linezolid or moxifloxacin and pyrazinamide in murine models of tuberculosis. Antimicrob Agents Chemother 63:e00021-19. doi:10.1128/AAC.00021-19 30833432 PMC6496099

[14] Li SY, Irwin SM, Converse PJ, Mdluli KE, Lenaerts AJ, Nuermberger EL. 2015. Evaluation of moxifloxacin-containing regimens in pathologically distinct murine tuberculosis models. Antimicrob Agents Chemother 59:4026–4030. doi:10.1128/AAC.00105-15 25918146 PMC4468727

[15] Tasneen R, Li S-Y, Peloquin CA, Taylor D, Williams KN, Andries K, Mdluli KE, Nuermberger EL. 2011. Sterilizing activity of novel TMC207- and PA-824-containing regimens in a murine model of tuberculosis. Antimicrob Agents Chemother 55:5485–5492. doi:10.1128/AAC.05293-11 21930883 PMC3232786

[16] Li SY, Tasneen R, Tyagi S, Soni H, Converse PJ, Mdluli K, Nuermberger EL. 2017. Bactericidal and sterilizing activity of a novel regimen with bedaquiline, pretomanid, moxifloxacin, and pyrazinamide in a murine model of tuberculosis. Antimicrob Agents Chemother 61:e00913-17. doi:10.1128/AAC.00913-17 28630203 PMC5571308

[17] Zhang M, Li SY, Rosenthal IM, Almeida DV, Ahmad Z, Converse PJ, Peloquin CA, Nuermberger EL, Grosset JH. 2011. Treatment of tuberculosis with rifamycin-containing regimens in immune-deficient mice. Am J Respir Crit Care Med 183:1254–1261. doi:10.1164/rccm.201012-1949OC 21330452 PMC3114054

[18] Lenaerts AJ, Chapman PL, Orme IM. 2004. Statistical limitations to the Cornell model of latent tuberculosis infection for the study of relapse rates. Tuberculosis (Edinb) 84:361–364. doi:10.1016/j.tube.2004.03.002 15525559

[19] Mourik BC, Svensson RJ, de Knegt GJ, Bax HI, Verbon A, Simonsson USH, de Steenwinkel JEM. 2018. Improving treatment outcome assessment in a mouse tuberculosis model. Sci Rep 8:5714. doi:10.1038/s41598-018-24067-x 29632372 PMC5890284

[20] Mudde SE, Ayoun Alsoud R, van der Meijden A, Upton AM, Lotlikar MU, Simonsson USH, Bax HI, de Steenwinkel JEM. 2022. Predictive modeling to study the treatment-shortening potential of novel tuberculosis drug regimens, toward bundling of preclinical data. J Infect Dis 225:1876–1885. doi:10.1093/infdis/jiab101 33606880 PMC9159334

[21] Berg A, Clary J, Hanna D, Nuermberger E, Lenaerts A, Ammerman N, Ramey M, Hartley D, Hermann D. 2022. Model-based meta-analysis of relapsing mouse model studies from the critical path to tuberculosis drug regimens initiative database. Antimicrob Agents Chemother 66:e0179321. doi:10.1128/AAC.01793-21 35099274 PMC8923195

[22] Walter ND, Born SEM, Robertson GT, Reichlen M, Dide-Agossou C, Ektnitphong VA, Rossmassler K, Ramey ME, Bauman AA, Ozols V, et al.. 2021. Mycobacterium tuberculosis precursor rRNA as a measure of treatment-shortening activity of drugs and regimens. Nat Commun 12:2899. doi:10.1038/s41467-021-22833-6 34006838 PMC8131613

[23] Dide-Agossou C, Bauman AA, Ramey ME, Rossmassler K, Al Mubarak R, Pauly S, Voskuil MI, Garcia-Cremades M, Savic RM, Nahid P, Moore CM, Tasneen R, Nuermberger EL, Robertson GT, Walter ND. 2022. Combination of Mycobacterium tuberculosis RS ratio and CFU improves the ability of murine efficacy experiments to distinguish between drug treatments. Antimicrob Agents Chemother 66:e0231021. doi:10.1128/aac.02310-21 35311519 PMC9017352

[24] Musisi E, Dide-Agossou C, Al Mubarak R, Rossmassler K, Ssesolo AW, Kaswabuli S, Byanyima P, Sanyu I, Zawedde J, Worodria W, Voskuil MI, Savic RM, Nahid P, Davis JL, Huang L, Moore CM, Walter ND. 2021. Reproducibility of the ribosomal RNA synthesis ratio in sputum and association with markers of Mycobacterium tuberculosis. Microbiol Spectr 9:e0048121. doi:10.1128/Spectrum.00481-21 34494858 PMC8557932

[25] Hendrix J, Mubarak RA, Bateman A, Massoudi LM, Rossmassler K, Kaya F, Zimmerman MD, Wynn EA, Voskuil MI, Robertson GT, Moore CM, Walter ND. 2025. Physiologic recovery of Mycobacterium tuberculosis from drug injury: a molecular study of post antibiotic effect in mice. BioRxiv. doi:10.1101/2025.02.25.640123 PMC13128115 42018569

[26] Rouan MC, Lounis N, Gevers T, Dillen L, Gilissen R, Raoof A, Andries K. 2012. Pharmacokinetics and pharmacodynamics of TMC207 and its N-desmethyl metabolite in a murine model of tuberculosis. Antimicrob Agents Chemother 56:1444–1451. doi:10.1128/AAC.00720-11 22155815 PMC3294950

[27] Irwin SM, Prideaux B, Lyon ER, Zimmerman MD, Brooks EJ, Schrupp CA, Chen C, Reichlen MJ, Asay BC, Voskuil MI, Nuermberger EL, Andries K, Lyons MA, Dartois V, Lenaerts AJ. 2016. Bedaquiline and pyrazinamide treatment responses are affected by pulmonary lesion heterogeneity in Mycobacterium tuberculosis infected C3HeB/FeJ mice. ACS Infect Dis 2:251–267. doi:10.1021/acsinfecdis.5b00127 27227164 PMC4874602

[28] Bustion AE, Ernest JP, Kaya F, Silva C, Sarathy J, Blanc L, Imperial M, Gengenbacher M, Xie M, Zimmerman MD, Robertson GT, Weiner D, Via LE, Barry CE, Savic RM, Dartois V. 2025. The kinetics of bedaquiline diffusion in tuberculous cavities open a window for emergence of resistance. J Infect Dis 232:e431–e441. doi:10.1093/infdis/jiaf303 40464735 PMC12455318

[29] Wynn EA, Dide-Agossou C, Al Mubarak R, Rossmassler K, Hendrix J, Voskuil MI, Obregón-Henao A, Lyons MA, Robertson GT, Moore CM, Walter ND. 2025. Deconvoluting drug interactions based on M. tuberculosis physiologic processes: transcriptional disaggregation of the BPaL regimen in vivo. Genomics. doi:10.1101/2025.02.24.639926 PMC12587609 40965512

[30] Chen C, Ortega F, Alameda L, Ferrer S, Simonsson USH. 2016. Population pharmacokinetics, optimised design and sample size determination for rifampicin, isoniazid, ethambutol and pyrazinamide in the mouse. Eur J Pharm Sci 93:319–333. doi:10.1016/j.ejps.2016.07.017 27473307

[31] Siefert HM, Domdey-Bette A, Henninger K, Hucke F, Kohlsdorfer C, Stass HH. 1999. Pharmacokinetics of the 8-methoxyquinolone, moxifloxacin: a comparison in humans and other mammalian species. J Antimicrob Chemother 43 Suppl B:69–76. doi:10.1093/jac/43.suppl_2.69 10382878

[32] Ahmad Z, Peloquin CA, Singh RP, Derendorf H, Tyagi S, Ginsberg A, Grosset JH, Nuermberger EL. 2011. PA-824 exhibits time-dependent activity in a murine model of tuberculosis. Antimicrob Agents Chemother 55:239–245. doi:10.1128/AAC.00849-10 20937781 PMC3019674

[33] Clary J, Roberts JK, Hanna D, Tagliavini A, Sordello S, Upton A, Hermann D, Al B. 2025. A stochastic simulation-based approach to inform relapsing mouse model (RMM) study design. bioRxiv. doi:10.1101/2025.07.18.665504 PMC12888889 41459930

