# Supplementary material for: A modeling-based framework to evaluate forgiveness of tuberculosis treatment in a BALB/c relapsing mouse model
Source: Antimicrob Agents Chemother. 2026 Jan 15;70(2):e01109-25. doi: 10.1128/aac.01109-25 (PMC12888910; doi:10.1128/aac.01109-25)
Supplement: Supplemental material — Tables S1, S2 and S3; Fig. S1 and S2. [file aac.01109-25-s0001.docx]

**A modeling-based framework to evaluate forgiveness of tuberculosis treatment in a BALB/c relapsing mouse model.**

**Supplementary data :**

|  | **Infection phase** | | **Treatment phase (Tx)** | | | | | | | | **Relapse Phase (R)** | | | | | | | |
| --- | --- | --- | --- | --- | --- | --- | --- | --- | --- | --- | --- | --- | --- | --- | --- | --- | --- | --- |
| **Treatment** | **Day -13**  **(1 dpi)** | **Day 0**  **(14 dpi)** | **end of**  **2W Tx** | **end of**  **4W Tx** | **end of**  **6W Tx** | **end of**  **8W Tx** | **end of**  **10W Tx** | **end of**  **12W Tx** | **end of**  **14W Tx** | **end of**  **16W Tx** | **2W Tx + 12WR** | **4W Tx + 12W R** | **6W Tx + 12W R** | **8W Tx + 12W R** | **10W Tx + 12W R** | **12WTx + 12W R** | **14W Tx + 12W R** | **16W Tx + 12W R** |
| **Untreated** | 5(CFU) | 5(CFU) + 3(RS) | 5 (RS) | - | - | - | - | - | - | - | - | - | - | - | - | - | - | - |
| **BPaMZ 7/7** | - | - | 5 (CFU) + 5(RS) | 5(CFU) + 5(RS) | 5(CFU) + 5(RS) | 5(CFU) +  3(RS) + 6(PK) | - | - | - | - | 6 | 6 | 6 | 6 | - | - | - | - |
| **BPaMZ 5/7** | - | - | 5(CFU) + 5(RS) | 5(CFU) + 5(RS) | 5(CFU) + 5(RS) | 5(CFU) +  3(RS) + 6(PK) | 5(CFU) | 5(CFU) | - | - | 6 | 6 | 6 | 6 | 6 | - | - | - |
| **2RHZE/ 2RH 7/7** | - | - | 5(CFU) + 5(RS) | 5(CFU) + 5(RS) | 5(CFU) + 5(RS) | 5(CFU) +  3(RS) + 6(PK) | 5(CFU) | 5(CFU) | 5(CFU) | 5(CFU) | - | - | - | 6 | 6 | 6 | 6 | 6 |
| **2RHZE/ 2RH 5/7** | - | - | 5(CFU) + 5(RS) | 5(CFU) + 5(RS) | 5(CFU) + 5(RS) | 5(CFU) +  3(RS) + 6(PK) | 5(CFU) | 5(CFU) | 5(CFU) | 5(CFU) | - | - | - | 6 | 6 | 6 | 6 | 6 |

**Table S1** **Treatment groups: number of mice per treatment group at each endpoint for each readout during the different phase of the study:**

Each phase, infection (day post infection: dpi) treatment (Tx) and Relapse (R) duration are indicated in week (W). the number of mice used per readout is indicated before the parameter measured in bracket (CFU, RS for RS ratio or PK). For the relapse only the number of mice used per time point is indicated for measuring the bacterial load at the end of 12 weeks off treatment (+12WR)

| **Compound** | **Transition** | **CV^1^** | **CE^2^** |
| --- | --- | --- | --- |
| Moxifloxacin | 402.18 > 110.08 | 22 | 34 |
| Bedaquiline | 557.16 > 229.15 | 50 | 22 |
| Pretomanid | 360.06 > 175.09 | 16 | 28 |
| Pyrazinamide | 123.98 > 80.99 | 50 | 16 |
| Rifampicin | 823.44 > 95.02 | 16 | 50 |
| Isoniazid | 138.01 > 78.99 | 22 | 22 |
| Ethambutol | 205.18 > 116.03 | 16 | 16 |
| BDQ-M2 | 541.10 > 480.11 | 34 | 22 |

^1^ CV: Cone Voltage

^2^ CE: Collision Energy

**Table S2: Liquid Chromatography –Mass Spectrometry (i.e. LC-MS) parameters**

| Compound | LOQ | | |
| --- | --- | --- | --- |
|  | Plasma (ng/mL) | Blood (ng/mL) | Lung (ng/g) |
| Bedaquiline | 6 | 6 | 250 |
| Pretomanid | 12 | 12 | 5 |
| Moxifloxicin | 6 | 6 | 5 |
| Pyrazinamide | 200 | 200 | 10 |
| B-M2 | 3 | 60 | 38500 |
| Rifampicin | 30 | 40 | 250 |
| Isoniazid | 40 | 300 | 250 |
| Pyrazinamide | 400 | 2000 | 200 |
| Ethambutol | 10 | 100 | 25 |

**Table S3: Bioanalysis Limit of Quantification (LOQ)**


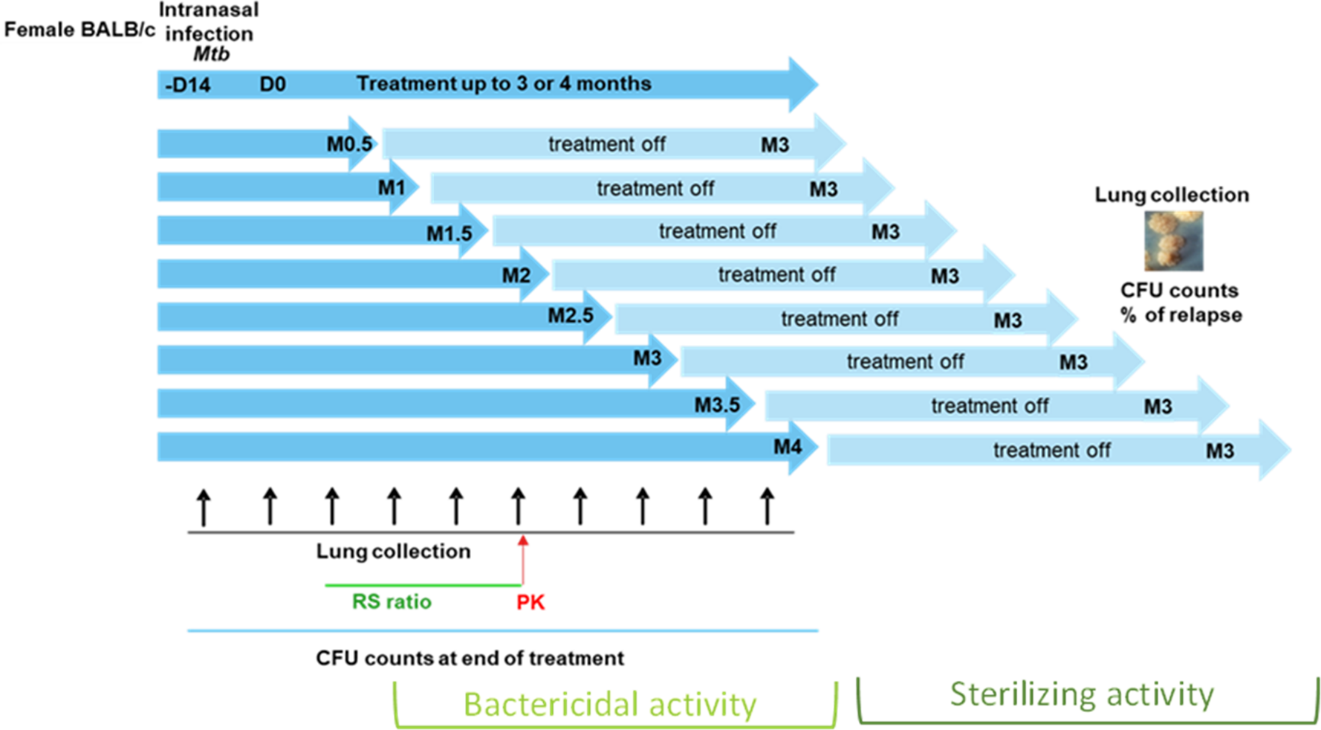


**Figure S1 Individual blood PK after 8 weeks of treatment on the fifth day of the last week of treatment**

After 8 weeks of treatment on the 5th day of the last week, for each combination (6 mice per group) blood was collected at 0.5, 2.5, 5.5, 7.5, 9 and 24 hours post-first compound dosing. Individual drug exposure was determined in BPaMZ (A) and RHZE (B) after 5/7 or 7/7 dosing. Of note: Dotted Line in graph corresponds to time of dosing start of corresponding drug.


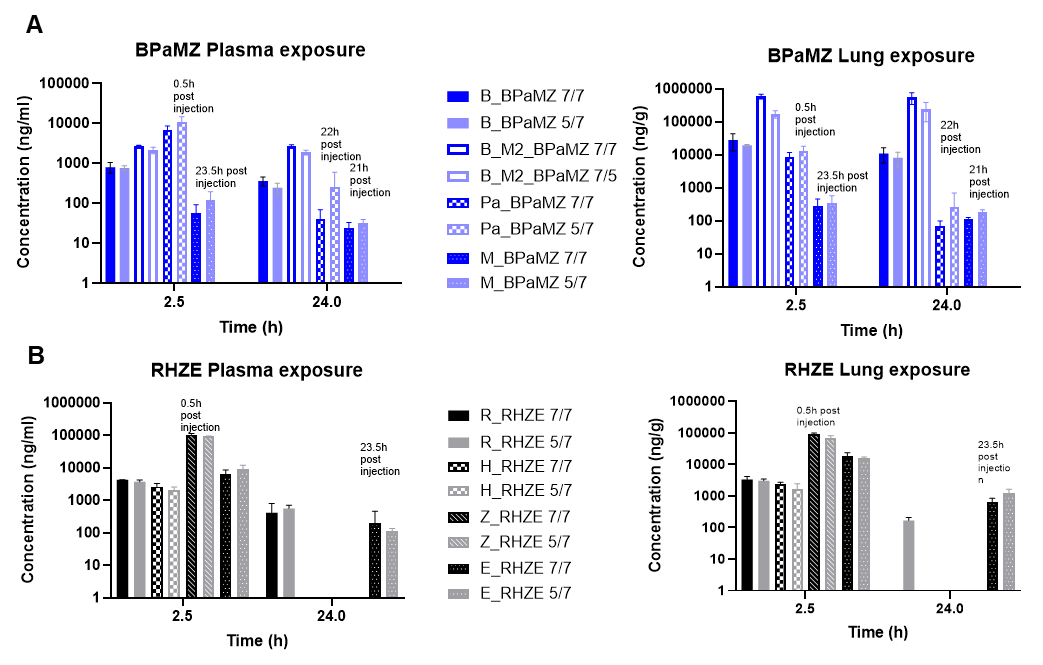


**Figure S2 Plasma and Lung exposure after 8 weeks of treatment the fifth day of the last week of treatment at 2.5h and 24h post dosing**

After 8 weeks of treatment, for each combination, at terminal time point, blood was collected by cardiac puncture for plasma preparation and lungs were collected at 2.5h and 24h post first compound of regimen. Individual drug exposure was determined in BPaMZ (A) and RHZE (B) after 5/7 or 7/7 dosing.
